# COVID-19–Associated *Fusobacterium nucleatum* Bacteremia, Belgium

**DOI:** 10.3201/eid2703.202284

**Published:** 2021-03

**Authors:** Louis Wolff, Delphine Martiny, Véronique Yvette Miendje Deyi, Evelyne Maillart, Philippe Clevenbergh, Nicolas Dauby

**Affiliations:** Université Libre de Bruxelles, Brussels, Belgium (L. Wolff, D. Martiny, V.Y. Miendje Deyi, E. Maillart, P. Clevenbergh, N. Dauby);; Saint-Pierre University Hospital Brussels, Brussels (L. Wolff, N. Dauby);; Université de Mons, Mons, Belgium (D. Martiny);; Laboratoire Hospitalier Universitaire de Bruxelles–Universitair Laboratorium Brussel, Brussels, (D. Martiny, V.Y. Miendje Deyi);; Brugmann University Hospital, Brussels (E. Maillart, P. Clevenbergh)

**Keywords:** Fusobacterium nucleatum, bacteremia, bacteria, tocilizumab, respiratory infections, severe acute respiratory syndrome coronavirus 2, SARS-CoV-2, SARS, COVID-19, coronavirus disease, zoonoses, viruses, coronavirus

## Abstract

We report 4 cases of *Fusobacterium nucleatum* bacteremia associated with coronavirus disease (COVID-19). Three cases occurred concomitantly with COVID-19 diagnosis; 1 occurred on day 15 of intensive care. None of the patients had known risk factors for *F. nucleatum* bacteremia. *F. nucleatum* infection could represent a possible complication of COVID-19.

*Fusobacterium nucleatum* is a gram-negative anaerobic rod member of the oral and digestive microbiota (*1*). *F. nucleatum* is an uncommon cause of bacteremia; annual reported incidence is 0.22–0.34 cases/100,000 population (*1*,*2*). Risk factors for *F. nucleatum* bacteremia include malignancy, older age, alcohol abuse, immunosuppression, and dialysis; infection is often hospital-acquired (*1*,*2*). Mortality rates for *F. nucleatum* bacteremia can reach 10% (*1*,*2*). 

In March and April 2020, 2 major hospitals in Brussels, Belgium, observed 4 cases of monomicrobial *F. nucleatum* bacteremia, all associated with severe acute respiratory syndrome coronavirus 2 (SARS-CoV-2) infection among patients with coronavirus disease (COVID-19). In contrast, the same hospitals reported a total of 4 *F. nucleatum* cases in 2019, 3 in 2018, 2 in 2017, 1 in 2016, and 2 in 2015. However, the hospital emergency plan initiated on March 14 during the first wave of the COVID-19 pandemic in Belgium prohibited all nonurgent medical care. Thus, the 2020 *F. nucleatum* incidence cannot be extrapolated and compared with previous years because of modifications of patient characteristics.

*F. nucleatum* was cultured from patients’ blood specimens by using a BD BACTEC FX blood culture system (Becton Dickinson, https://www.bd.com) and pure isolates were successfully identified by using matrix-assisted laser desorption/ionization time-of-flight mass spectrometry (Bruker Daltonics, https://www.bruker.com). Cross-contamination was formally excluded because blood cultures became positive on different days and bacterial identifications were performed on separate sets of experiments (Table 1). 

Nasopharyngeal swab samples were collected from the 4 patients. Three patients tested positive for SARS-CoV-2 by reverse transcription PCR (RT-PCR) using the RealStar SARS-CoV-2 RT-PCR kit (Altona Diagnostics, https://www.altona-diagnostics.com) and 1 by a COVID-19 Ag Respi-Strip rapid antigen test (Coris Bioconcept, https://www.corisbio.com). All 4 patients had concomitant pneumonia compatible with COVID-19 on chest computed tomography (CT) scans. The patients were 34, 51, 52, and 70 years of age (median 51.5 years); the median age was lower than in previously reported *F. nucleatum* bacteremia (*1*,*2*), but the sample size is too small for statistical analysis. None of the patients had any classical risk factors for *F. nucleatum* bacteriemia. The youngest patient had no underlying conditions. Three patients had abdominal symptoms and 2 underwent abdominal CT with contrast, but both had unremarkable results. Three patients had symptoms of bacteremia at the time of COVID-19 diagnosis; bacteremia was diagnosed in the other patient after 15 days in the hospital intensive care unit (ICU). The ICU patient received a single 800-mg intravenous dose of tocilizumab (TCZ) to treat COVID-19–associated hyperinflammatory syndrome. Increased risk for severe infection, including bacteremia, has been associated with long-term TCZ treatment when administered for non-COVID-19 indications (*3*). To our knowledge, no previous *F. nucleatum* infection has been reported with TCZ use in general. The patient died of COVID-19–related severe respiratory failure on day 21 in the ICU, but the other 3 patients were discharged to home without complications. 

Although SARS-CoV-2 infection initially was described as an agent of severe pneumonia, other organ involvements are now well described. Other studies among hospitalized COVID-19 patients have shown that 18%–48% had digestive complaints ranging from anorexia to diarrhea and abdominal pain (*4*,*5*). RT-PCR detected the virus in the feces of 48%–53% of patients with abdominal complaints and feces remained positive in 20%–33% of patients even after respiratory samples converted from RT-PCR–positive to negative (*4*,*6*). The propensity of SARS-CoV-2 to infect digestive organs might be explained by the fact that angiotensin converting enzyme 2, a known receptor used by the virus to enter human cells, has been found to be highly expressed in enterocytes (*4*,*7*). 

The reservoir of *F. nucleatum* is generally considered to be the oral cavity (*8*). Only 1 of these patients had oral symptoms, but no oral lesions were observed. The 3 other patients had abdominal symptoms, suggesting that bacteremia might be the consequence of translocation from the digestive tract (*9*). *F. nucleatum* has been shown to colonize colon mucus with associated mucosal inflammation (*10*). 

In conclusion, digestive tract invasion by SARS-CoV-2 and secondary inflammatory response might promote translocation of opportunistic pathogens, such as *F. nucleatum*, and further research could elucidate this interaction. Nonetheless, our observations suggest that anaerobe bacteremia should be considered as a complication of COVID-19. 

**Table:** Characteristics of 4 cases of *Fusobacterium nucleatum* bacteremia in patients with COVID-19, Belgium*

Characteristic	Patient 1	Patient 2	Patient 3	Patient 4
Age, y	52	51	34	70
COVID-19 diagnosis	Day of admission	Day of admission	Day of admission	Day of admission
Symptoms at diagnosis	Dry cough and sore throat for 7 d	Cough, abdominal pain, and diarrhea for 7 d	Cough, abdominal pain, and diarrhea for 7 d	Fever (38.5°C), vomiting for 1 d
Underlying conditions	Hypertension	Diabetes, hypertension, obesity	None	Diabetes, hypertension, hypothyroidism, history of stroke
Radiological findings				
Chest CT	Ground glass opacities in all lobes	Diffuse infiltrates in all lobes	Ground glass opacities in 10% of lungs	Ground glass opacities in 10% of lungs
Abdominal CT with contrast	NA	NA	Unremarkable	Unremarkable
Blood culture collection	Day 1	Day 15	Day 1	Day 1
Time to positivity (no. sets)	96 h (1 of 2)	55 h (1 of 2)	72 h (1 of 2)	72 h (1 of 2)
COVID-19 therapy	HCQ 400 mg oral 2×/d on day 1, then 200 mg 2×/d for 4 d	HCQ 400 mg oral 2×/d on day 1, then 200 mg 2×/d for 4 d; TCZ 800 mg IV once; and RDV 200 mg IV loading dose, then 100 mg 4×/d for 4 d	None	None
Antimicrobial drug therapy	None	TZP 4 g IV 4×/d for 6 d	MTZ 500 mg orally 3×/d for 7 d	None
*F. nucleatum* antimicrobial susceptibility testing				
Amoxicillin/clavulanic	S	S	S	S
Clindamycine	S	S	S	S
Imipenem	S	S	S	S
Metronidazole	S	S	S	S
Piperacilline/tazobactam	S	S	S	S
Outcome	Discharged home	Died	Discharged home	Discharged home

*COVID-19, coronavirus disease; CT, computed tomography; HCQ, hydroxychloroquine; IV, intravenous; MTZ, metronidazole; RDV, remdesivir; S, susceptible; TCZ, tocilizumab; TZP, piperacilline-tazobactam.
